# SAVER: sodium valproate for the epigenetic reprogramming of high-risk oral epithelial dysplasia—a phase II randomised control trial study protocol

**DOI:** 10.1186/s13063-021-05373-8

**Published:** 2021-07-05

**Authors:** Caroline McCarthy, Joseph Sacco, Stefano Fedele, Michael Ho, Stephen Porter, Triantafillos Liloglou, Bill Greenhalf, Max Robinson, Bridget Young, Silvia Cicconi, Seema Chauhan, Binyam Tesfaye, Richard Jackson, Frances Sherratt, Richard Shaw

**Affiliations:** 1 Department of Oral Medicine, Liverpool University Dental Hospital, Pembroke Place, Liverpool, L3 5PS UK; 2 Institute of Systems, Molecular and Integrative Biology, The University of Liverpool, Crown Street, Liverpool, L69 3BX UK; 3 University College London, UCL Eastman Dental Institute and NIHR UCLH Biomedical Research Centre, 21 University Street, London, WC1E 6DE UK; 4 Leeds Teaching Hospitals NHS Trust, Oral and Maxillofacial Surgery, Leeds Dental Institute, Clarendon Way, Leeds, LS2 9LU UK; 5 University College London, UCL Eastman Dental Institute, 21 University Street, London, WC1E 6DE UK; 6 GCP Laboratory Facility, Molecular and Clinical Cancer Medicine, University of Liverpool, 3rd Floor UCD Block, Duncan Building, Daulby Street, Liverpool, L69 3GA UK; 7 Dept of Cellular Pathology, Royal Victoria Infirmary, Queen Victoria Road, Newcastle upon Tyne, NE1 4LP UK; 8 Department of Psychological Sciences, Institute of Psychology, Health and Society, University of Liverpool, Whelan Building, Brownlow Hill, Liverpool, L69 3GB UK; 9 Liverpool Clinical Trials Centre, Block C, Waterhouse Building, 1-3 Brownlow Street, Liverpool, L69 3GL UK; 10 Liverpool Health Partners SPARK, 1st Floor IC3, Liverpool Science Park, 131 Mount Pleasant, Liverpool, L3 5TF UK; 11 Liverpool Clinical Trials Centre, University of Liverpool, 1st Floor, Mersey Bio, Liverpool, L69 7ZB UK; 12 Department of Public Health, Policy and Systems, University of Liverpool, B209, 2nd Floor Block B, Waterhouse Building, 1-5 Dover Street, Liverpool, L3 5DA UK; 13 Liverpool Head and Neck Centre; Institute of Systems, Molecular and Integrative Biology, The University of Liverpool, 200 London Road, Liverpool, L3 9TA UK

**Keywords:** Oral epithelial dysplasia, Sodium valproate, Epigenetic reprogramming, Drug repurposing, Randomised controlled trial, Chemoprevention

## Abstract

**Background:**

Sodium valproate (VPA) has been associated with a reduced risk of head and neck cancer development. The potential protective mechanism of action is believed to be via inhibition of histone deacetylase and subsequent epigenetic reprogramming. SAVER is a phase IIb open-label, randomised control trial of VPA as a chemopreventive agent in patients with high-risk oral epithelial dysplasia (OED). The aim of the trial is to gather preliminary evidence of the clinical and biological effects of VPA upon OED and assess the feasibility and acceptability of such a trial, with a view to inform a future definitive phase III study.

**Methods:**

One hundred and ten patients with high-risk OED will be recruited from up to 10 secondary care sites in the UK and randomised into either VPA or observation only for 4 months. Women of childbearing potential will be excluded due to the teratogenic properties of VPA. Tissue and blood samples will be collected prior to randomisation and on the last day of the intervention/observation-only period (end of 4 months). Clinical measurement and additional safety bloods will be taken at multiple time points during the trial. The primary outcome will be a composite, surrogate endpoint of change in lesion size, change in grade of dysplasia and change in LOH profile at 8 key microsatellite regions. Feasibility outcomes will include recruitment targets, compliance with the study protocol and adverse effects. A qualitative sub-study will explore patient experience and perception of the trial.

**Discussion:**

The current management options for patients with high-risk OED are limited and mostly include surgical resection and clinical surveillance. However, there remains little evidence whether surgery can effectively lead to a notable reduction in the risk of oral cancer development. Similarly, surveillance is associated with concerns regarding delayed diagnosis of OED progressing to malignancy. The SAVER trial provides an opportunity to investigate the effects of a repurposed, inexpensive and well-tolerated medication as a potential chemopreventive strategy for patients with high-risk OED. The clinical and biological findings of SAVER will inform the appropriateness, design and feasibility of a definitive phase III trial.

**Trial registration:**

The trial is registered with the European Clinical Trials Database (Eudra-CT 2018-000197-30). (http://www.isrctn.com/ISRCTN12448611). The trial was prospectively registered on 24/04/2018.

**Supplementary Information:**

The online version contains supplementary material available at 10.1186/s13063-021-05373-8.

## Background

The incidence of oral squamous cell carcinoma (OSCC) has been increasing for several decades and is predicted to rise 33% by 2035 [1]. The disease results in notably high rates of mortality and morbidity, with 50–60% 5-year survival [2]. Most oral cancers are preceded by long-standing clinical changes of the oral mucosa, mainly white (leukoplakia) and red (erythroplakia) patches, with progression from normal epithelium to invasive OSCC occurring through sequential stages of histological intra-epithelial changes including mild, moderate, and severe dysplasia. A recent systematic review suggests that OED is associated with oral cancer development in 12.1% of cases, with severe OED showing higher rates of progression compared to mild and moderate dysplasia (24.1% vs 10.3%) [3]. Management of OED should aim at reducing the risk of oral cancer development; however, there remains little convincing evidence that this can be achieved with any of the treatment strategies currently adopted in clinical practice [3–5]. OED is typically managed through surveillance or surgical resection [4]; however, neither have strong evidence to support their use nor address the underlying pathogenesis. Surveillance is associated with concerns regarding delayed diagnosis of OED progressing to OSCC [2]. Surgery is not always possible for all lesions or all patients, and recurrence rates for premalignant lesions range from 4 to 30% [6–9]. Also, localised therapies fail to treat the wider mucosal field, often encompassing the entire upper aerodigestive tract, and therefore do not address the risk of multifocal synchronous or metachronous dysplastic lesions. Accordingly, there is an urgent unmet need to develop well-designed clinical trials of novel interventions that, especially in high-risk OED, could effectively reduce the risk of progression to malignancy.

This paper presents the protocol for the SAVER trial, a multi-site, phase IIb, open-label, randomised control trial of VPA in high-risk OED. This trial offers treatment to patients with high-risk lesions who may otherwise have no other option for active treatment and addresses the need for further research in the area of chemoprevention treatment in OED [10]. This protocol report adheres to SPIRIT guidance (Standard Protocol Items: Recommendations for Interventional Trials) [11].

### Evidence supporting the use of sodium valproate as a chemopreventive agent for head and neck cancer

The Kang study is a cohort study of approximately 440,000 patients in the US Veterans’ Affairs System, with long-term psychiatric or neurological diagnoses and at increased risk of smoking-related cancers; 26,000 had been taking VPA for more than one year [12]. There was a lower incidence of head and neck malignancy in the group taking VPA, with a 32% protective effect reported (HR 0.68, 95% CI 0.50–0.93). The reduction in risk was maintained in a multivariate analysis for age, sex, race, past vs current smoking, psychiatric or neurological disease, chronic obstructive pulmonary disease and alcohol and substance use (HR 0.66, 95% CI 0.48–0.92). The weight of this observation is reinforced by dose effect, with both the length of treatment and dose of VPA correlating with a further reduction of risk. The most plausible mechanism of reduction of cancer risk is through the epigenetic reprogramming effects of VPA through histone deacetylase (HDAC) inhibition.

### Rationale for the SAVER trial

The unmet need for an effective treatment to prevent progression of OED to oral cancer, the risk-reduction effect demonstrated in the Kang study and the underlying plausible epigenetic mechanisms underpin the need for a clinical trial using VPA as an HDAC inhibitor in the chemoprevention of oral cancer in individuals with high-risk OED. HDACis are an emerging class of drugs that have shown promise as anticancer agents when used alone or in combination with conventional therapies. HDACi and VPA have been comprehensively reviewed in their role in combination therapies, with either cytotoxic chemotherapy or targeted agents, in haematological malignancy or recurrent/metastatic solid tumours [13, 14]. There is good evidence for clinical benefit of epigenetic therapy in haematological pre-malignancies, such as myelodysplastic syndromes [15]. In contrast, the rationale for HDACi monotherapy in the setting of chemoprevention for oral lesions presents a differing opportunity and there is a paucity of data. There is no in vivo data related to HDACi in oral carcinogenesis; however, there is in vitro evidence of the effect of HDACi on growth inhibition and mediation of apoptosis of OSCC cell lines [16–18]. There is also evidence of growth suppression of xenograft tumours in nude mice [19].

Before embarking in a long and expensive phase III trial, we suggest that preparatory research is needed in order to (i) inform important aspects of the trial design such as feasibility and outcome measures and (ii) identify a preliminary signal of clinical efficacy linked to reasonable biological mechanisms. We have therefore designed a phase IIb trial so to gather data on such preliminary evidence of effect, explore outcome measures and assess the feasibility and acceptability of the trial in this population.

It is expected that the clinical, mechanistic and feasibility data of SAVER will inform the decision for a future larger phase III trial with robust cancer (cancer development) endpoints, necessitating much larger cohorts and longer follow-up.

## Methods and analysis

### Study design and objectives

The SAVER trial is a phase IIb multicentre, open-label, randomised control trial of VPA vs observation only in patients with high-risk OED (see Table 1). The aim of this trial is to investigate the effects of VPA as an epigenetic chemopreventive therapy in high-risk OED. The primary objective is to determine: clinical activity of sodium valproate in high-risk OED, using a composite surrogate endpoint of change in the size of clinical lesion, changes in loss of heterozygosity profile and change in grade of dysplasia (see the ‘Outcome measures’ section). The key secondary objectives are to determine, in subjects with high-risk OED: the mechanism of action of VPA, associated toxicity, overall survival, development of any cancer and the feasibility of conducting such research in the UK National Health Service.

**Table 1:** Definition of a high-risk lesion

Definition of a high-risk lesion:	
The index lesion is deemed at high risk of malignant transformation (i.e. estimated >20% over 5 years) if:	
a. Diagnosed as severe OED (WHO grade) or;	
b. Diagnosed as mild or moderate OED (WHO grade), with at least one additional high-risk feature(s) from the list below:	
i. Patient is a non-smoker (less than 100 cigarettes or equivalent over whole lifetime)	
ii. Lesion size > 200 mm^2^	
iii. Lateral tongue site	
iv. Mucosal speckling or heterogeneous appearance	
v. Patient had an excised OSCC during previous 5 years (but not within previous 6 months).	

### Recruiting centres

SAVER is coordinated by the Liverpool Clinical Trials Centre (LCTC) and is sponsored by the University of Liverpool.

### Study population and inclusion criteria

Patients will be recruited via Oral Medicine, Oral and Maxillofacial Surgery outpatient and Oral Dysplasia Multidisciplinary clinics at the study sites. Eligible patients will have a lesion that is accessible, measurable (at least 100 mm^2^), amenable to clinical photography and located in the oral cavity or oropharynx or on the lip. As part of the screening process for SAVER, all patients will require an incisional biopsy of the index lesion to confirm eligibility; only patients with a biopsy confirming a histopathological diagnosis of OED, considered to be at high risk of transforming to oral cancer, will be included. The definition of a high-risk lesion can be found in Table 1.

There must be a treatment plan for either surgical resection or close monitoring (clinical and photographic follow-up). The patient must be fully informed, have received Patient Information Sheet (PIS) and considered this during a ‘cooling-off’ period, be competent to consent, age ≥ 18 and able to comply with minimum attendance requirements. Exclusion criteria can be found in Table 2.

**Table 2:** Exclusion criteria for the SAVER trial

**Exclusion criteria**	
1. Synchronous or metachronous OSCC (i.e. at time of screening or within 6 months)	
2. Active malignancy outside head and neck region (with exception of non-melanoma skin cancer)	
3. Currently positive for COVID-19 (patients who have recovered from COVID-19 are NOT excluded)	
4. OSCC susceptible conditions e.g. Fanconi anaemia, Blooms syndrome, ataxia-telangectasia and Li-Fraumeni syndrome	
5. Clinical and/or histopathological diagnosis of oral submucous fibrosis	
6. Immunosuppression. However, low dose i.e. < 10mg/day prednisolone, or equivalent steroid, (as per BNF conversion table), are not considered an exclusion.	
7. Chronic previous or current use of sodium valproate	
8. Diagnosed epilepsy that has chronic previous or current use of *any* antiepileptic therapy	
9. Obesity (body mass index ≥ 30)	
10. Known relative or absolute contraindications to Sodium Valproate (as listed in British National Formulary), and specifically:	
a. Acute porphyria	
b. Known or suspected mitochondrial disorders	
c. Personal or family history of severe hepatic dysfunction, current hepatic dysfunction (as evidenced by LFTs outwith reference range and prolonged prothrombin time)	
d. Past history or current pancreatitis	
e. Women with child-bearing potential. A woman is considered of childbearing potential (WOCBP), i.e. fertile, following menarche and until becoming post-menopausal unless permanently sterile. Permanent sterilisation methods include hysterectomy, bilateral salpingectomy, and bilateral oophorectomy.	
f. Potential drug interactions (particularly antipsychotic and anticonvulsant medications, MAO inhibitors, antidepressants, benzodiazepines), specifically patients taking phenobarbital, primodone, carbopenem antibiotics (imipenem, panipenem, meropenem), cimetidine, erythromycin, lamotrigine, olanzapine, pivmecillinam, sodium oxybate, zidovudine, carbamazepine, phenytoin, rifampicin, high dose salicylates including aspirin > 75 mg daily (patients taking low dose aspirin 75 mg daily are eligible).	
g. Patients with suicidal ideation and behaviour should be excluded from the trial. Patients should also be monitored for signs of suicidal ideation and behaviours and appropriate treatment should be considered.	
h. Patients with known or suspected mitochondrial disease, systemic lupus erythematosus or hyperammonaemia	

Details of the management of patients being randomised or having surgery within the SAVER trial during the COVID-19 pandemic can be found in Additional file 1.

### Randomisation

Randomisation will be completed centrally by the LCTC via a password-protected web-based tool called the Treatment Allocation Randomisation System (TARDIS). All patients will be allocated a unique randomisation number. Randomisation must be carried out within 90 days of the research biopsy report and commencement of trial treatment within 30 days of the date of randomisation. Patients will be randomised between VPA (arm A) and observation only (arm B) in the ratio 2:1, with site used as a stratification factor.

#### Arm A

VPA (Epilim) will initially be taken orally for 14 days at a dose of 500 mg once daily. From day 15 until 4 calendar months after day 1, VPA will be taken orally continuously at a dose of 500 mg twice daily. A 5-mm punch biopsy will be taken at the 4-month time point.

#### Arm B

Patients in the control group will not receive any trial medication. They will be monitored at the same intervals as patients in Arm A (2, 4 and 6 months from baseline) and will subject to the same blood monitoring schedule. A 5-mm punch biopsy will be taken at the 4-month time point, as for Arm A.

### Schedule of events

**Fig. 1:**
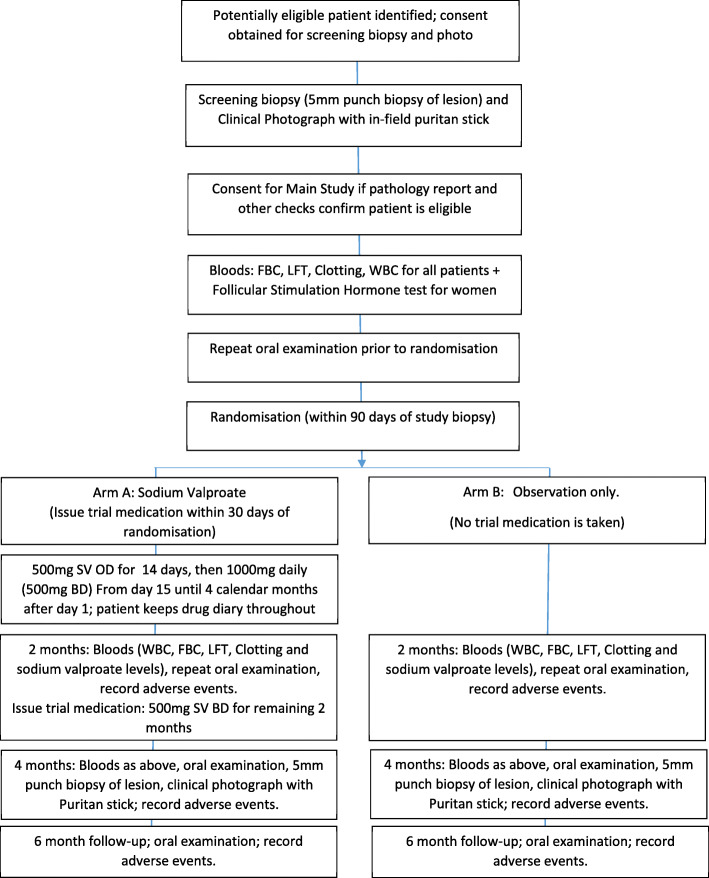
Patient activity through the SAVER trial

**Fig. 2:**
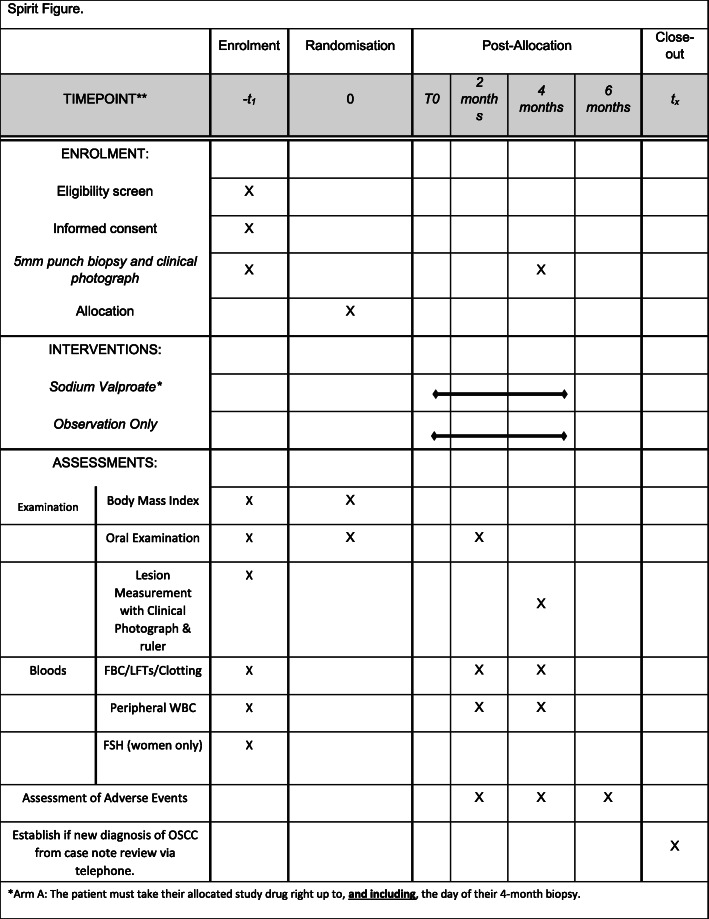
Spirit figure

Figure 1 shows the flow of patients through the trial and the SPIRIT figure (Fig. 2) gives details of the procedures required at each trial time-point. A screening biopsy (incisional biopsy) is taken to confirm eligibility, prior to randomisation. Patients are randomised to one of two arms (sodium valproate or observation only), for 4 months. A repeat biopsy is taken on the final day of trial medication at the 4-month time-point for both groups. A clinical photograph and bloods are also taken at this visit for both groups. In total the patient is examined five times during the trial period for signs of lesion progression.

Adverse events are monitored through the Liverpool Adverse Events Reporting Questionnaire at 3 points during the trial and at any unscheduled trial visits. All patients will have a full blood count, clotting screen and LFTs performed as outlined in the schedule, to assess for potential haematological and hepatic toxicities and clotting aberrations.

### Clinical progression during the trial

If a lesion is seen to have progressed and the clinician is concerned regarding malignant transformation, an urgent biopsy is requested and reported through the central pathology unit at Newcastle Hospital. If the diagnosis confirms malignant transformation, the patient ceases study drug (if relevant) is withdrawn from the trial and returns to normal clinical management and follow-up. If histopathology does not show malignant transformation, the patient returns to normal study schedule or normal standard of care.

## Outcome measures

### Primary outcome measure

Response to treatment will be measured using a surrogate endpoint. It is not feasible to use ‘malignant transformation’ as an endpoint in this context, as a large, long-term study would be required to allow sufficient time for enough events to occur for statistical power. The primary endpoint itself will be derived as a composite score of changes in lesion size, changes in histological grade and loss of heterozygosity (LOH), which is advised in the context of rare events.

#### Assessment of lesion size

Lesion size will be calculated based on the estimated elliptical area given by the longest length of the lesion and the associated perpendicular width. Lesion size response will be then measured on a 7-point scale ranging from − 3 to 3 based on the change in lesion size between pre and post-treatment assessment (Table 3).

**Table 3:** Scale for measuring change in the size of clinical lesion

Change in lesion size	Code
≥ 75% decrease	3
50–74 % decrease	2
25–49% decrease	1
0–24% decrease or increase	0
25–49% increase	− 1
50–74% increase	− 2
≥ 75% increase	− 3

#### Assessment of histology response score

A 9-point scale will be used to obtain the histological score as related to the grade of dysplasia. The histology response score is the original pathology score minus the final pathology score (from the specimen obtained at the end of the 4-month treatment period). See Table 4.

**Table 4:** Histological scoring scale

Score	Histological diagnosis
0	Normal with or without hyperkeratosis
1	Atypia with crisply defined clinical margins
2	Mild dysplasia
3	Mild-moderate dysplasia
4	Moderate dysplasia
5	Moderate-severe dysplasia
6	Severe dysplasia
7	Carcinoma in situ
8	Invasive squamous cell carcinoma

#### Assessment of loss of heterozygosity (LOH) response score

LOH has been identified as a prognostic marker for progression of OED, with lesions displaying LOH at 3p and 9p having a 22-fold increased risk of progression to OSCC [20]. The following 8 loci and associated genes have been selected based on previous evidence of their predictive value in the progression of OED:

- 3p14 [D3S1007 (VHL), D3S1234 (FHIT)]
- 9p21 [D9S171, D9S1748 (P16/CDKN2A), D9S1751 (P16)]
- 9p22 (IFN-a)
- 17p13 [D17S786 (P53) and TP53]

For each loci, a score of +1 is given if it is positive for LOH and zero if it is negative for LOH. The LOH response score will be the pre-treatment score minus the post-treatment score.

#### Total responsiveness score

The total responsiveness score for each patient will be calculated as follows: lesion size response score + histological response score + LOH response score. Patients will be classified as ‘disease progression’ with a response score ≤ − 1, ‘stable disease’ with a response score between − 1 and 1 and ‘response to treatment’ with a response score ≥ 1. The only exception to these criteria is for patients with confirmed malignant transformation. These patients shall automatically be categorised as having disease progression, irrespective of their responsiveness score. The disease response rate will compare patients with response to treatment against patients with either stable disease or disease progression.

### Secondary outcome measure

Secondary endpoints will be assessed including toxicity (measured using Common Terminology Criteria for Adverse Events (CTCAE) v4.03 classification), overall survival and malignancy of the head and neck (or other sites) during the patient’s active trial period and for the total duration of the trial. Feasibility endpoints will be recorded, defined by overall and centre-specific rate of recruitment, compliance with treatment, protocol deviations and drop-out rates.

### Mechanistic sub-study

VPA has a known mechanism of action as a histone deacetylase inhibitor (HDACi). The reduced risk of head and neck cancers demonstrated in patients taking VPA has been hypothesised to be through epigenetic reprogramming of oral potentially malignant disorders [12]. As part of an embedded mechanistic sub-study, we will assess tissue-specific epigenetic changes, changes in gene expression, expressed markers of proliferation, apoptosis and senescence. We will also assess pharmacodynamic biomarkers of histone acetylation in circulating white cell DNA, from venous blood samples taken at the 2- and 4-month time points. The presence of a control group will enable us to determine whether these effects are specific to VPA. Tissues samples will be obtained before and after drug treatment. Samples will be bisected and half used for histology and immunohistochemistry and half used for RNA/DNA preparation.

### Qualitative sub-study: the SAVER information study

This is the first UK-based chemoprevention trial in OED, therefore we will explore patients’ perspectives on the acceptability of SAVER and their experience of recruitment and participation in the trial. This will be achieved through 20 qualitative interviews, or until data saturation is reached. The aim is to improve patient information resources and to enhance the design and acceptability of a future phase III trial. We will interview patients who declined to participate in the trial and those who took part; we will aim for diversity in key characteristics: demographics, trial site and proposed treatment (surgery vs surveillance).

### Sample size calculation

Sample size calculations are carried out on the principles of a single-stage Jung design for randomised phase II studies based on exact binomial probabilities and allowing for unequal allocation [21]. The primary outcome is the response rate; a rate of 20% is estimated for the control arm with a 20% rate increase in the treatment arm representing the minimal clinically important difference. With a type I error of 0.16 and 82% of power, 100 patients (33 in the observation-only arm and 67 in the treatment arm) will be required in the study. Adjusting for a potential 10% drop-out rate, the final sample size will be of 110 patients (37 in the observation-only arm and 73 in the treatment arm).

The intent of the study will be to recruit the 110 patients required over a period of 32 months, assuming 10 sites recruiting at an average rate of 0.4 patients per site per month.

### Blinding

SAVER is an open-label trial. The primary endpoint of the trial consists of objective assessments of lesion size using a clinical photograph, grade of dysplasia and loss of heterozygosity studies. These will all be assessed by blinded assessors.

### Statistical analysis

There will be no formal stopping rules or interim analysis based on patients’ response rate. However, an Independent Safety and Data Monitoring Committee (ISDMC) will meet at regular intervals to review and assess the conduct of the study and the accumulated data. The ISDMC will be able to make appropriate recommendations to the Trial Steering Committee (TSC) on grounds of toxicity and feasibility.

The study will be analysed and reported in line with the Consolidated Standards of Reporting Trials (CONSORT). Statistical analyses will be carried out following the intention to treat principle (ITT), with the exception of toxicity which will be analysed on the basis of the actual treatment received. Missing data are not anticipated to be substantial in the study and therefore the final analysis shall be performed on a complete case basis. However, if more than 10% missing data are observed on the primary outcome, multiple imputation technique by chained equations shall be used.

Continuous variables will be summarised as median and interquartile range and categorical variables will be presented as frequencies of counts with associated percentages. Primary analysis on the primary outcome will be performed using a stratified Mantel Haenszel test and results will be assessed with a one-sided p value of 0.16 as the threshold for statistical significance. The primary efficacy parameter (odds ratio) will also be reported with the one-sided 84% confidence interval. Logistic regression will be used to investigate the relationship of the primary outcome with key prognostic covariates. Analyses of categorical secondary outcome will mirror that of response rate, using stratified Mantel Haenszel test and logistic regression techniques. Time to event data will be compared between arms by stratified log-rank test. Further analyses on survival data shall be carried out using Cox models if, after inspection of Schoenfeld residuals, the proportional hazard assumption is not violated. All secondary analyses will be assessed by the nominal two-sided p value of 0.05 to determine statistical significance.

### Ethical considerations

The trial protocol has been reviewed and approved by North West - Haydock Research Ethics Committee and REC reference: 18/NW/0180. The trial has been registered with the MHRA; the ISRCTN number is 12448611.

### Sodium valproate safety profile

Sodium valproate is licensed for use in epilepsy and bipolar disorder. It is also used off-label for depression, neuropathic pain, dementia and migraine. Very common adverse effects, defined as occurring in >10% of patients, include nausea and tremor. Common adverse events (between 1 and 10% of patients) include upper abdominal cramps, transient increase in liver enzymes, weight gain and diarrhoea, transient alopecia, reduced bone density, thrombocytopenia and anaemia. Neurological adverse effects such as fatigue, sedation, confusion and dizziness are also observed commonly. Due to the significant risk of birth defects and developmental disorders in babies born to mothers taking VPA during pregnancy, the MHRA have issued guidance advising: ‘Valproate medicines must no longer be used in women or girls of childbearing potential unless a Pregnancy Prevention Programme is in place’. Women of childbearing potential (WOCBP) will not be recruited to the trial, due to known teratogenic effects of VPA [22]. All women will require a follicular stimulating hormone (FSH) test prior to randomisation, to confirm post-menopausal status. The exception to this is women who have undergone total hysterectomy or bilateral salpingo-oophorectomy.

The SAVER trial will use VPA at 1000 mg/day; this is a low to medium dose, associated with mild or absent toxicities, and is well tolerated [23]. Higher doses, sometimes justified in epilepsy, are associated with weight gain, tremor, drowsiness and cognitive slowing. In the context of premalignant oral conditions, we feel that these would not be justified. The impact of weight gain will be reduced by excluding obese patients (BMI > 30).

#### Potential risks of delay to surgical treatment

Study participants who are listed for surgical excision of OED will have their surgery scheduled at the end of month 4 of the study. This is considered an acceptable timeframe as the progression of OED to invasive cancer is reported to take an average of 4 years from diagnosis [3]. It is also in keeping with common clinical practice at most HNC services in the UK, which prioritise treatment of invasive oral cancer [24].

### Criteria for discontinuing or modifying allocated interventions

Patients may be withdrawn from treatment for any of the following reasons: Development of an OSCC, unacceptable toxicity, any change in the patient’s medical condition that justifies discontinuation of treatment in the clinician’s opinion and pregnancy.

Treatment may be discontinued for any toxicity with a significant impact on quality of life (generally grade 2 or higher; however, persistent grade 1 adverse events (AEs) may also lead to discontinuation). A 50% dose reduction (500 mg/day) may also be considered for persistent grade 1 toxicities rather than withdrawal from the trial. Patients discontinuing due to toxicity will be followed up and assessed as per protocol. Patients discontinuing treatment for any reason will continue to be followed up unless the patient explicitly withdraws consent for follow-up. If the patient explicitly states their wish not to contribute further data to the study, a withdrawal CRF will be completed. Anonymised data collected up to this point can still be used for study purposes.

### Assessment of compliance with study treatment

All patients will have plasma valproate levels measured at 2 months and 4 months; this will confirm that patients in the observation-only arm are not taking sodium valproate and will help to confirm patient compliance in the treatment arm. In addition, patients in the treatment arm will be given a drug diary sheet to be completed each day. Research Nurses will collect the unused tablets and completed diaries and record any circumstances of non-compliance in the patient notes and on the CRF. The returned medication will be sent to the site pharmacy for storage.

### Recruitment

The main feasibility outcome is the recruitment rate. Ten sites have been selected to allow an achievable recruitment rate of 0.4 patients per site per month (approximately 4 patients per month) with a total of 110 patients. Targets for recruitment will be set at 43 and 88 for 12 and 24 months respectively. As a guide, it is proposed that if the study will be recruiting within 80% of the intended rate (at least 34 and 70 for 12 and 24 months, respectively) then no action will be taken. If the study is recruiting between 50% and 80% (between 22–33 and 44–69 for 12 and 24 months, respectively) of the intended rate, the ISDMC may recommend continuation only if strategies will be put in place to increase recruitment. If the study is recruiting at less than 50% (less than 22 and 44 for 12 and 24 months, respectively) of the intended rate, the ISDMC may recommend early termination of the study on the grounds of feasibility.

### Adverse events

All adverse events that occur from the point of the patient’s written informed consent are to be reported, even if the patient has not started taking VPA. All adverse events should be reported up to the point of the primary endpoint being established, with the exception of malignant transformation or new head and neck cancer, which should be collected until trial closure. All non-serious adverse events (AE)/adverse reactions (AR), whether expected or not, will be recorded in the relevant page of the CRF. Serious adverse reactions (SARs), serious adverse events (SAEs) and suspected unexpected serious adverse reactions (SUSARs) should be reported within 24 h of the local site becoming aware of the event. The SAE form asks for the nature of event, date of onset, severity, corrective therapies given, outcome and causality. The responsible investigator should sign the causality of the event. Additional information should be sent within 5 days if the reaction has not resolved at the time of reporting. The LCTC will notify the MHRA and main Research Ethics Committee (REC) of all SUSARs occurring during the study according to the following timelines; fatal and life-threatening within 7 days of notification and non-life-threatening within 15 days. All investigators will be informed of all SUSARs occurring throughout the study. Local investigators should report any SUSARs and/or SAEs as required by their Local Research Ethics Committee and/or Research and Development Office.

### Confidentiality

Individual participant medical information obtained through this study is considered confidential and disclosure to third parties is prohibited. Case report forms will be labelled with patient initials and unique trial randomisation number. Tissue samples will be transferred to both the pathology and GCLP laboratories and will be identifiable by unique trial randomisation number only. Consent forms sent to the LCTC as part of the randomisation process may contain patient identifiers for monitoring as described in the trial risk assessment. Such information will be stored separately from the patient folders in secure, locked cabinets. Each participating site will maintain appropriate medical and research records for this trial, in compliance with the International Council for Harmonisation of Technical Requirements for Registration of Pharmaceuticals for Human Use (ICH) E6 Good Clinical Practice (GCP), Section 4.9 and regulatory and institutional requirements for the protection of confidentiality of subjects.

### Qualitative sub-study: the SAVER information study

Audio-recordings of semi-structured interviews will be transferred to a professional transcription agency, with a legally binding confidentiality agreement, via a secure upload facility. Completed transcripts will be checked by the qualitative researcher on receipt and pseudo-anonymised ready for analysis. Audio recordings of the interviews will be retained in case of queries until the end of the study at which point the recordings will be destroyed.

### Audit

The SAVER investigational sites, facilities, laboratories and all data (including sources) and documentation must be available for GCP audit and inspection by competent or independent ethics committees and the LCTC. Such audits/inspections may take place at any sites where trial-related activity is taking place (i.e. the Sponsor site(s), LCTC or at any investigators site, including laboratories, pharmacies etc.). The site staff shall assist in all aspects of audit or inspection. The LCTC has an internal audit programme which will be produced annually by the LCTC Quality Assurance (QA) Manager. The LCTC operate a risk-based audit programme, based on the assessment of risks associated with both processes with the quality system and the specific trials.

### Data collection and data management

Trial data for SAVER will be captured using an electronic case report form (eCRF), with the exception of randomisation, AEs and SAEs which will be processed on paper CRF. The SAVER Data Management Plan Data provides detailed information on data entry, coding, security and storage arrangements. The LCTC is housed in a building that is secured by swipe card access for authorised personnel and is locked when unattended. Documents are kept in locked cabinets.

### Protocol amendments

Plans for communicating important protocol modifications are detailed in LCTC’s document ‘Making Substantial and Non-substantial amendments.’ All protocol amendments will be communicated with the Sponsor, funder, research sites, patient representatives and all other key stakeholders as agreed in trial-specific contracts. Protocol amendments will only be implemented at research sites following the receipt of the necessary regulatory approvals. The LCTC ensures the receipt of amendment documentation from individual research sites.

### Dissemination and access to data

The results of this trial will be submitted for publication in relevant peer-reviewed publications and a plain English version, co-edited by patients, will be circulated to all trial participants. Results (including negative results) will be presented at national and international conferences. We do not have formal plans to share anonymised individual participant-level data.

### Ancillary and post-trial care

NHS Trust and Non-Trust Hospitals have a duty of care to patients treated, whether or not the patient is taking part in a clinical trial, and they are legally liable for the negligent acts and omission of their employees. Compensation is therefore available in the event of clinical negligence being proven.

## Roles and responsibilities

### Trial oversight and regulatory arrangements

#### Trial Management Group (TMG)

This comprises the chief investigator, other lead investigators (clinical and non-clinical), patient representative and members of the LCTC. The TMG will be responsible for the day-to-day running and management of the trial and will meet at least three times per year.

#### Independent Safety and Data Monitoring Committee (ISDMC)

The independent Safety and Data Monitoring Committee (ISDMC) consists of an independent chairperson in a related area of expertise, plus 2 independent members, one of whom is also an expert in a related area and another who is an expert in medical statistics. The ISDMC will be responsible for reviewing and assessing recruitment, interim monitoring of safety and effectiveness, trial conduct and external data. The ISDMC will first convene before the trial opens to recruitment and will then define frequency of subsequent meetings (at least annually). The ISDMC will provide a recommendation to the TSC concerning the continuation of the study.

#### Trial Steering Committee (TSC)

The Trial Steering Committee will consist of an independent chairperson, other independent experts in the field of oral cancer, a statistician and at least one patient representative. The role of the TSC is to provide overall supervision for the trial and provide advice through its independent Chairman. The ultimate decision for the continuation of the trial lies with the TSC.

##### Sponsorship

SAVER is sponsored by the University of Liverpool and coordinated by the LCTC in the University of Liverpool. The trial Sponsor has authority in all aspects of trial activities.

##### Registration

The SAVER trial is registered with the European Clinical Trials Database (Eudra-CT 2018-000197-30)

##### Clinical trial suthorisation

The trial has received clinical trial authorization by the Medicines and Healthcare Products Regulatory Authority (MHRA) under the Medicines For Human Use (Clinical Trials) Regulations 2004: 04196/0048/001-0001.

## Discussion

This study protocol describes a multi-site, open-label randomised control trial of VPA vs observation only in the management of high-risk OED. There is an unmet need for additional strategies in the management of OED. Patients are currently offered surgery or close surveillance, neither of which have sufficient supporting evidence for the prevention of malignant transformation of OED. Efforts in chemoprevention of malignant transformation of OED have been limited and this represents the first trial of VPA as a chemopreventive agent in OED. The SAVER trial will investigate the clinical activity of VPA, using a composite endpoint of clinical, pathological and molecular changes. The mechanism of action will be explored using gene expression studies, investigation of epigenetic changes and expression of markers of cell cycle regulation. Feasibility of recruitment and patient perception of this type of trial will be assessed through interviews of patients approached for recruitment in the hope of informing the design of a future Phase III clinical trial.

## Trial Status

The SAVER trial is open (with a temporary halt to recruitment due to the COVID-19 pandemic). The current Protocol Version Number is 9 (date: March 2021). Recruitment began on 17/12/2019 and the study is due to complete in July 2023.

## Supplementary Information

**Additional file 1.** COVID-19 SAVER policy.

## Data Availability

Results from this trial will be published in an open access journal. A plain English version, co-edited by patients, will be circulated to all trial participants. Results will be presented at national and international conferences. We do not have formal plans to share anonymised individual participant-level data.

## Abbreviations

AE: Adverse event
AR: Adverse reaction
CRF: Case report form
FBC: Full blood count
GCLP: Good Clinical Laboratory Practice
GCP: Good Clinical Practice
HDAC(i): Histone deacetylase (inhibitor)
ICH: International Council for Harmonisation of Technical Requirements for Registration of Pharmaceuticals for Human Use
ISDMC: Independent Safety and Data Monitoring Committee
LCTC: Liverpool Clinical Trials Centre
LFT: Liver function tests
LOH: Loss of heterozygosity
MHRA: Medicines and Healthcare products Regulatory Agency
NHS: National Health Service (UK)
NIHR: National Institute for Health Research
OED: Oral epithelial dysplasia
OMFS: Oral and maxillofacial surgery
OSCC: Oral squamous cell carcinoma
PIS: Patient Information Sheet
QA: Quality assurance
REC: Research Ethics Committee
SAE: Serious adverse event
SAR: Serious adverse reaction
SPIRIT: Standard Protocol Items Recommendations for Interventional Trials
SUSAR: Suspected unexpected serious adverse reaction
TMG: Trial Management Group
TSC: Trial Steering Committee
VPA: Sodium valproate
WBC: White blood cells (peripheral)
WOCBP: Women of childbearing potential
