# Supplementary material for: SAVER: sodium valproate for the epigenetic reprogramming of high-risk oral epithelial dysplasia—a phase II randomised control trial study protocol
Source: Trials. 2021 Jul 5;22:428. doi: 10.1186/s13063-021-05373-8 (PMC8256209; doi:10.1186/s13063-021-05373-8)
Supplement: Supplementary file 1 — Additional file 1. COVID-19 SAVER policy. [file 13063_2021_5373_MOESM1_ESM.docx]

## COVID-19 SAVER policy

For patients undergoing surgery as part of the SAVER protocol, the following precautions are now mandated, as a minimum, in line with recommendations from the Royal College of Surgeons (England), data from the COVIDsurg cohort studies [20], and ENT-UK [21]. These requirements will apply to those having definitive resection of lesions at 4 months under general anaesthetic and will not be relevant to patients being managed with surveillance.

1. All patients recruited to SAVER should have their surgery in a designated COVID-Free environment, as defined by local service configuration and clinical practice.
2. COVID-19 PCR swab testing should take place 24 to 72 hours prior to surgery. The operating surgeon is responsible for ensuring the test result is negative before embarking on surgery.
3. Surgery should only proceed if the swab test is negative and the patient is asymptomatic for COVID-19 and apyrexial on the day of surgery.

Peri-operatively and post-operatively, the risk of COVID-19 infection to patients and staff should be minimised, through use of appropriate Personal Protective Equipment (PPE) by healthcare workers, implementing best practices and limiting visitors to the ward, as per local standard protocols.

Patients having local anaesthetic biopsy as part of the SAVER protocol are not subject to such strict criteria. For these procedures, patients are not required to undertake PCR testing and correspondingly are not managed in a designated COVID-free environment as these are minor outpatient attendances. For such procedures, it is assumed that local guidelines will ensure that biopsy should only proceed if the patient is asymptomatic for COVID-19 and apyrexial on the day of surgery. Appropriate PPE should be used through this procedure.

#### Patients who test positive for COVID-19 on the SAVER study:

Patients who test positive on SARS-CoV-2 PCR swab testing 24-72hrs prior to any surgery / biopsy or randomisation must have their surgery or randomisation delayed for at least 2 weeks or until full recovery from any associated illness (whichever is later) and also in line with local protocols. An SAE SAE form stating that the patient was ‘positive for COVID-19’ should be submitted. Patients who test positive for COVID-19 on trial, after randomisation should continue on the study subject to ongoing assessment of their medical fitness. An SAE form should be completed as above.
